# Power of Feedback-Induced Beta Oscillations Reflect Omission of Rewards: Evidence From an EEG Gambling Study

**DOI:** 10.3389/fnins.2018.00776

**Published:** 2018-10-30

**Authors:** Zachary Yaple, Mario Martinez-Saito, Nikita Novikov, Dmitrii Altukhov, Anna Shestakova, Vasily Klucharev

**Affiliations:** ^1^Centre for Cognition and Decision Making, Institute for Cognitive Neuroscience, National Research University Higher School of Economics, Moscow, Russia; ^2^Department of Psychology, National University of Singapore, Singapore, Singapore; ^3^Laboratory of Cognitive Psychophysiology, National Research University Higher School of Economics, Moscow, Russia; ^4^Faculty of Computer Science, National Research University Higher School of Economics, Moscow, Russia; ^5^MEG Center, Moscow State University of Pedagogical and Education, Moscow, Russia

**Keywords:** beta oscillations, EEG, time-frequency analysis (TFA), reward, risky decision making, feedback, gain omission, prediction error

## Abstract

The functional role of high beta oscillations (20–35 Hz) during feedback processing has been suggested to reflect unexpected gains. Using a novel gambling task that separates gains and losses across blocks and directly compares reception of monetary rewards to a ‘no-reward/punishment’ condition with equal probability we aimed to further investigate the role of beta oscillations. When contrasting different feedback conditions across rewards, we found that a late low beta component (12–20 Hz) had increased in power during the omission of rewards relative to the reception of rewards, while no differences were observed during the loss domain. These findings may indicate that late low beta oscillations in the context of feedback processing may respond to omission of gains relative to other potential outcomes. We speculate that late low beta oscillations may operate as a learning mechanism that signals the brain to make future adequate decisions. Overall, our study provides new insights for the role of late low beta oscillations in reward processing.

## Introduction

Effective decision-making crucially relies on the ability to improve decisions based on the evaluation of feedback. A learning (e.g., prediction-error) signal is computed after observing the outcome generated by each choice which are used to improve the quality of future decisions (Rangel et al., 2008). There is growing interest in the neural mechanisms associated with the processing of rewarding feedback. Using electroencephalography (EEG), many studies attempt to explore the neural mechanisms of feedback learning by examining neural oscillations. Specifically, many have attempted to explore the functional role of high beta oscillations (20–35 Hz) between 200 and 400 ms which tend to increase in oscillatory power in response to monetary gains compared to monetary losses (Marco-Pallerés et al., 2008).

A further exploration in a follow up experiment demonstrated that beta oscillations tend to respond to rare rewarding events. When comparing cued gain and loss incentives with high or low probability, beta oscillations were stronger in power when the probability of cued rewards had a low relative to a high probability (HajiHosseini et al., 2012), suggesting that feedback-related beta oscillations are sensitive to the reception of *unexpected* gains (Marco-Pallerés et al., 2015). However, a further attempt to explore this hypothesis reveal no association between probability of outcome, expected value nor reward prediction errors manifested by beta band rhythm (HajiHosseini and Holroyd, 2015b).

Perhaps one possible drawback to the above-mentioned studies is that monetary gains are typically compared directly with monetary losses, neither of which may serve as an adequate baseline (Proudfit, 2015) since gains and losses typically induce neural activity of distinct neural networks (see Mohr et al., 2010 for meta-analysis). Therefore, the functional role of beta oscillations is still open for debate and further investigation is necessary (see Luft, 2014 for review).

For this study we aimed to investigate the functional role of beta oscillations by comparing gains and losses separately with a “no gain” and a “no loss” feedback condition. To achieve this goal, we employed a novel risky decision-making task that allows one to compare the reception and omission of monetary incentives separately for gains and losses (Yaple et al., 2017, 2018). This task requires participants to select between risky and safe options, depending on whether to switch or repeat task-sets.

The purpose of this task design was to further explore the functional role of beta oscillations using a risky decision-making paradigm with gains and losses portrayed across blocks. Rather than manipulating probability between trials, this paradigm will allowed us to compare feedback associated with uncertain (risk) outcomes relative to feedback produced from certain (safe) outcomes separately across gains and losses. Based on the prior study that shown how frontal beta oscillations respond to reward valence but not probability (HajiHosseini and Holroyd, 2015b), we predicted a change in power within the high beta range specifically for gains.

We aimed to compare feedback display representing the reception and omission of gains and losses with an uncertain outcome (±50 monetary units [MU] with a probability of 50%), relative to a certain outcome (±25 MU with a probability of 100%). Moreover, using trial by trial analysis we aim to test whether power of beta oscillations may predict risky decision making. Together our analyses will allow us to explore whether beta oscillations are responsive to gain or loss events and to assess whether this signal may facilitate decisions in upcoming trials.

## Materials and Methods

### Participants

Twenty-five healthy participants (23 right-handed; 18 females; mean age 21.61; age range 18–34 years; and *SD* = 4.49) with normal or corrected to normal vision and with no neurological disorders participated in the study. All participants provided a written consent approved by The Higher School of Economics Committee on Interuniversity Surveys and Ethical Assessment of Empirical Research in accordance with the Declaration of Helsinki. All participants were screened for psychological/psychiatric disorders and none of them reported use of drugs or alcohol in the days preceding the experiment.

### Stimuli and Procedure

**FIGURE 1:**
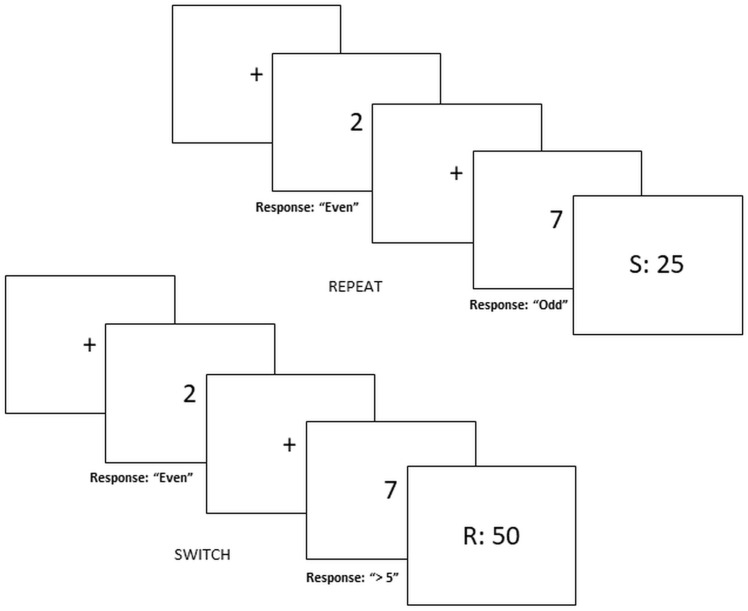
Switch-risk task. Risky decision making depends on voluntary switching and repeating task-sets. Safe decisions yield 25 MU with a probability of 100% whereas risky decisions yield 50 MU or 0 MU with a probability of 50%. Figure represents trial in the “Switch = Risk” reward block.

We used a novel risky decision-making task – “rewarded voluntary switch task” (see Yaple et al., 2017 for original design). It combines the voluntary task switching paradigm (Arrington and Logan, 2004, 2005; Arrington et al., 2014) with a two-choice financial decision-making task (e.g., Selten et al., 1999; Engelmann and Tamir, 2009; Harrison et al., 2013). This task requires participants to select between risky or safe gambles, depending on whether to voluntarily switch or repeat task-sets (i.e., an Odd/Even game or a Higher/Lower game). The additional rule of switching/repeating task sets is to ensure that participants are motivated to attend to task rules. See Figure 1 for visual representation of the paradigm.

Each trial begins with a centered fixation cross displayed between 500 and 1000 ms followed by a screen containing a single digit (1, 2, 3, 4, 6, 7, 8, or 9). To select between *risky* and *safe* decisions participants must select one out of two task-sets represented as an *Odd/Even game* or a *Higher/Lower than 5 game* by pressing one of the corresponding buttons (odd, even, high, and low). For half of the blocks, *repeating* the same game in successive trials would yield the *safe decision* while *switching* between game types would yield a *risky decision*. In the other half of the experiment, instructions are then counterbalanced such that switching results in a safe decision and repeating leads to a risky decision.

Gains and losses are separated across blocks. During gain blocks, participants are instructed that safe decisions are defined as “100% probability that you would receive 25 monetary units (MU),” while risky decisions were defined as “50% probability that you would receive 50 MU” (or alternatively 0 MU). In loss blocks, the safe decision are defined as “100% probability that you would lose 25 MU” while risky decisions are defined as “50% probability that you would lose 50 MU” (alternatively 0 MU).

For each response a feedback screen displayed for 2000 ms indicated the amount of MUs rewarded or lost for that particular trial. Positive feedback in gain blocks was 50 and 0 MU for loss blocks. Negative feedback in gain blocks was 0 MU and -50 MU for loss blocks. Neutral feedback was 25 and -25 MU for gain and loss blocks, respectively. For risky choices, a random generator displayed positive or negative feedback such that the distribution of feedback type was not fixed but randomly assigned. If response time exceeded 4000 ms or participants responded erroneously participants viewed negative feedback (e.g., 0 MU for gain block, -50 MU for loss blocks).

The experiment was programmed using E-Prime 2.0 software. Stimuli were centered on the screen and remained on the screen until a response was made. The text was displayed in black font on a gray scale background and all participants were instructed to use both hands. Participants received two rounds of training, which consisted of eight blocks of 10 trials, resulting in 80 trials in total. If accuracy was below 95% additional training sessions were provided. This learning phase was reflected in the actual experiment; accuracy for all except one participant (86%) was above 92%.

Initially, nine participants received 16 blocks of 30 trials (480 trials total). Due to the notion that probability of feedback did not vary between trials, choices varied between subjects, and that a substantial amount of trials were removed from the analysis, a potential problem with the analysis may be due to too few trials. Therefore, the number of trials per block was increased to 40 trials (640 trials total) for the remaining sixteen participants. After performing the task, participants were shown the total cumulative feedback on the computer screen. Participants received 500 MU for participation (500 MU ≈ 7 USD) and an additional bonus, between -300 and + 300 MU, based on the feedback outcomes of six randomly selected trials to maintain an equal motivation for risky decision making across blocks (see Krajbich et al., 2012).

### EEG Recording

The EEG data were recorded with BrainAmp amplifiers and BrainVision Recorder software (Brain Products GmbH, Munich, Germany) using silver ActiCap active scalp electrodes mounted in an elastic cap located at 60 standard positions according to the international 10–20 system. The electrophysiological signals were filtered online using a sampling rate of 500 Hz in the frequency range 0.2–100 kHz. Impedances were kept <10 kΩ. Electrooculogram were recorded with electrodes placed at both lateral canthi and below the left eye. EEG signals were referenced to the mean of the activity at the two mastoid processes.

Data preprocessing of the EEG data was performed using BrainVision Analyzer 2.0. First, signals in bad channels were replaced using nearest-neighbor interpolation. Second, a bandpass filter (0.1–40 Hz) was applied to the data, after which eye-blink- and eye-movement-related activity was suppressed in the data using independent component analysis. Finally, intervals containing non-systematic artifacts produced by electromyographic activity, skin potentials and other sources were manually rejected from the data. Across subjects, 10.1% (σ = 0.090) of trials were excluded from the analysis. For the first group, the mean number of trials excluded from the analysis was 15.6% (σ = 0.123); for the second group 8.0% (σ = 0.061) of trials were excluded. The mean number of valid trials included in EEG analysis across each condition for all subjects was 80.7 (range: 66–101 trials). The range of trials removed from each group was: 0–40 (from 480 trials) and 0–63 (from 640 trials).

### Time-Frequency Power Analysis

EEG analysis for each *feedback* (positive, neutral, and negative) x *valence* (gain, loss) condition was performed using Brainstorm (Tadel et al., 2011), which is documented and freely available for download online under the GNU general public license^1^.

Single trial time-frequency analysis was performed on a time window between -1000 and 2000 ms for each condition. For each trial, the segmented EEG data was convolved with a complex Morlet wavelet (from 1 to 40 Hz, linear increase). The frequency and time resolution of were set at the default settings (temporal resolution of 3 s at frequency 1 Hz) in Brainstorm, which uniquely define the temporal and spectral resolution of the wavelet for all other frequencies (Tadel et al., 2011). Changes in time varying energy (i.e., event-related spectral perturbations: (x-μ) / (μ^∗^100)) with respect to pre-stimulus baseline (-200 to -1 ms) were computed per condition and averaged for each subject.

### Statistical Analysis

Response times of risky and safe decisions were analyzed across gains and losses using a repeated measures analysis of variance (ANOVA) and a Bonferroni correction procedure. To determine whether the percentage of selected risky gambles was above or below chance level (μ = 50%), a one sample *t*-test across all conditions was computed. In addition, a paired sample *t* test was computed to assess whether percentage of selected risky gambles differed between gain and loss blocks. Cohen’s d was used as the calculation for effect size for one-sample and paired *t* tests; partial eta squared was used to estimate effect size for the ANOVA tests. Incorrect trials and trials in which participants responded longer than 4000 ms were excluded from the analysis.

Mean beta power (12–20 Hz) was calculated for FCz, FC1, FC2, Cz, C1, C2, CPz, CP1, and CP2 electrode positions for each feedback and valence condition within the 700–1000 ms post-response time window and entered into a repeated measures ANOVA test. Greenhouse-Geisser correction was applied.

The selection of the specific frequency band, latency interval, and electrode positions was purely data-driven and based on statistical analysis of the ERSP data averaged over the experimental conditions (the analysis was orthogonal to our main analysis). The ERSP data was tested against zero using permutational statistics on t-score maps transformed with the TFCE (threshold-free cluster enhancement) algorithm (see Novikov et al., 2015, 2017 for prior examples; also see Smith and Nichols, 2009). For more details, see Supplementary Materials. The analysis was performed using a custom-written Matlab scripts (The MathWorks, Inc.).

### *Post hoc* Testing

To assess whether the spectral power density of beta frequency influenced risky decisions in the following trial, we included several generalized linear models (GLMs) with a logit link function, performed separately for gains and losses. Spectral power density is characterized by the distribution of power for each frequency range within a specified time series (Duff et al., 2008). Predictors for these models included: positive feedback (with neutral feedback as reference), negative feedback (with neutral feedback as reference), beta (12–20 Hz), and theta (4–8 Hz) power spectral density. To compare these results, we also computed two GLMs with negative feedback as the reference variable corresponding with gains and losses. Theta power spectral density was included in the first two models to control for frequency specificity of beta (Tables 1, 2), yet in further analysis we also computed GLMs excluding theta power as a predictor (see Supplementary Tables 1, 2), corresponding to neutral and negative feedback as the reference variable, respectively. Wald tests (Kuznetsova et al., 2016) were performed on all levels up to 2 interactions. Analysis of the GLMs were performed using R software (R Core Team, 2016) with the software package lme4 (Bates et al., 2014) and lmertest (Kuznetsova et al., 2016). Family-wise error rate was controlled using a Holm-Bonferonni correction procedure.

**Table 1:** Generalized Logistic Model (GLM) predicting risk decision making in the following trial for rewards **(A)** and losses **(B)** with neutral feedback as the reference variable.

	β	*SE*	*z*-value	*p*-value	*p*′
**(A) GLM for rewards with neutral feedback as reference**					
Theta PSD	-0.155	0.058	-2.672	0.007	0.063
Beta PSD	0.094	0.046	2.025	0.042	0.378
**Fb (+50)**	**0.246**	**0.066**	**3.693**	**2.2 × 10**^-^**^4^**	**0.002**
**Fb (+0)**	**0.271**	**0.066**	**4.065**	**4.8 × 10**^-^**^5^**	**4.32 × 10**^-^**^4^**
Theta^∗^Beta PSD	0.019	0.025	0.756	0.449	>0.999
Theta PSD^∗^Fb (+50)	0.112	0.069	1.616	0.106	0.954
Theta PSD^∗^Fb (+0)	0.008	0.069	0.124	0.901	>0.999
**Beta PSD^∗^Fb (+50)**	-**0.390**	**0.080**	-**4.883**	**1.05 × 10**^-^**^6^**	**9.45 × 10**^-^**^6^**
Beta PSD^∗^Fb (+0)	-0.130	0.074	-1.751	0.079	0.711
**(B) GLM for losses with neutral feedback as reference**					
Theta PSD	-0.050	0.056	-0.891	0.372	>0.999
Beta PSD	0.061	0.052	1.184	0.236	>0.999
**Fb (-0)**	**0.606**	**0.658**	**9.197**	**<2 × 10**^-^**^16^**	**1.8 × 10**^-^**^15^**
**Fb (-50)**	**0.494**	**0.066**	**7.438**	**1.02 × 10**^-^**^13^**	**9.18 × 10**^-^**^13^**
Theta^∗^Beta PSD	0.024	0.025	0.977	0.328	>0.999
Theta PSD^∗^Fb (-0)	0.123	0.074	1.654	0.098	0.882
Theta PSD^∗^Fb (-50)	-0.079	0.078	-1.020	0.307	>0.999
Beta PSD^∗^Fb (-0)	-0.197	0.080	-2.453	0.014	0.126
Beta PSD^∗^Fb (-50)	-0.116	0.072	-1.601	0.109	0.981

*Spectral power density was extracted from each trial between 4 and 8 Hz (Theta PSD) and 12 and 20 Hz (Beta PSD). Bold font indicates statistical significance after Holm-Bonferroni correction. β, Beta coefficient represent standardized effect sizes; SE, Standard error of the mean; z-value based on Wald test; PSD, Power Spectral Density; *p*′, corrected p value; and Fb, Feedback.*

**Table 2:** Generalized Logistic Model (GLM) predicting risk decision making in the following trial for rewards **(A)** and losses **(B)** with negative feedback as the reference variable.

	β	*SE*	*z*-value	*p*-value	*p*′
**(A) GLM for rewards with negative feedback as reference**					
Theta PSD	-0.146	0.061	-2.365	0.018	0.162
Beta PSD	-0.035	0.067	-0.519	0.603	>0.999
Fb (+50)	-0.025	0.071	-0.357	0.721	>0.999
**Fb (+25)**	-**0.271**	**0.066**	-**4.065**	**4.8 × 10**^-^**^5^**	**4.32 × 10**^-^**^4^**
Theta^∗^Beta PSD	0.019	0.025	0.756	0.449	>0.999
Theta PSD^∗^Fb (+50)	0.103	0.072	1.426	0.154	>0.999
Theta PSD^∗^Fb (+25)	-0.008	0.069	-0.124	0.901	>0.999
**Beta PSD^∗^Fb (+50)**	-**0.260**	**0.092**	-**2.828**	**0.004**	**0.036**
Beta PSD^∗^Fb (+25)	0.130	0.074	1.751	0.079	0.711
**(B) GLM for losses with negative feedback as reference**					
Theta PSD	-0.130	0.065	-1.979	0.047	0.423
Beta PSD	-0.054	0.070	-0.776	0.437	>0.999
Fb (-0)	0.111	0.071	1.555	0.120	>0.999
**Fb** (-**25**)	-**0.494**	**0.066**	-**7.438**	**1.02 × 10**^-^**^13^**	**9.18 × 10**^-^**^13^**
Theta^∗^Beta PSD	0.024	0.025	0.977	0.328	>0.999
Theta PSD^∗^Fb (-0)	0.203	0.083	2.439	0.014	0.126
Theta PSD^∗^Fb (-25)	0.079	0.078	1.020	0.307	>0.999
Beta PSD^∗^Fb (-0)	-0.081	0.089	-0.915	0.360	>0.999
Beta PSD^∗^Fb (-25)	0.116	0.072	1.601	0.109	0.981

*Spectral power density was extracted from each trial between 4 and 8 Hz (Theta PSD) and 12 and 20 Hz (Beta PSD). Bold font indicates statistical significance after Holm-Bonferroni correction. β, Beta coefficient represent standardized effect sizes; SE, Standard error of the mean; z-value based on Wald test; PSD, Power Spectral Density; p′, corrected p value; and Fb, Feedback.*

### Source Analysis

For the beta frequency component, source localization for each feedback condition across gains and losses were performed on single trials between 12 and 20 Hz between the 700–1000 ms time window. A default anatomy of the standard MNI brain was used to compute a head model using OpenMEEG software (Gramfort et al., 2010) with a symmetric boundary element model as an EEG forward model of volume currents. Prior to source-localization, a noise covariance matrix was calculated based on the prestimulus interval between -500 and 0 ms to estimate the level of noise among the electrodes. Cortically unconstrained source-localization was performed on each trial using the standardized low resolution brain electromagnetic tomography (LORETA) technique. For each subject we calculated sources using a low spatial resolution of 2000 vertices and projected the grand averages to 15000 vertices to increase spatial resolution for the images. Resulting source maps per subject were averaged across trials for each condition. For visualization purposes, the source activation maps were thresholded to only show activations of 10 adjacent vertices or more with at least 40% of the maximum amplitude.

## Results

### Behavioral Analysis: Descriptive Statistics

**FIGURE 2:**
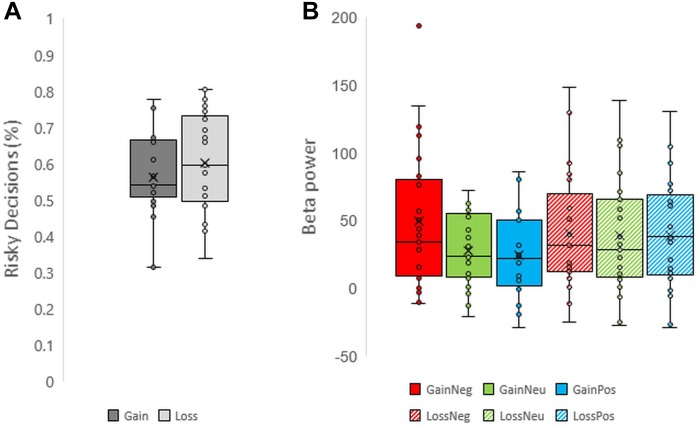
Boxplots representing **(A)** risky decision making across valence and **(B)** beta power across each condition for each individual.

Participants performed the task correctly: mean accuracy was 96.7% (σ = 0.029). Overall subjects preferred risky decisions (58.2%, σ = 0.121) more often than safe decisions (*p* = 0.003, Cohen’s *d* = 0.672; one sample *t*-test). Additional one sample *t*-tests on risky decisions also revealed a significant preference for risky decisions compared to safe decisions for both gains (60.34% risk) and losses (56.29% risk; See Figure 2A for individual scores of risky decision-making). The number of risky decisions across gains and losses blocks differed; the proportion of risky decisions was significantly greater in loss blocks compared to gain blocks (*p* = 0.020, Cohen’s *d* = 0.398; paired sample *t*-test), indicating a trend to select risky decisions more in loss blocks compared to gain blocks. On average participants responded more slowly when they selected safe decisions than risky decisions (*F*_1,24_ = 9.566, *p* = 0.005, and partial η^2^ = 0.285) and more slowly in the loss condition compared to the gain condition (*F*_1,24_ = 17.867, *p* < 0.001, and partial η^2^ = 0.427). The interaction effect of *valence* and *decision* on reaction times was not significant (*p* = 0.690).

### Beta Power: Time-Frequency Analysis

**FIGURE 3:**
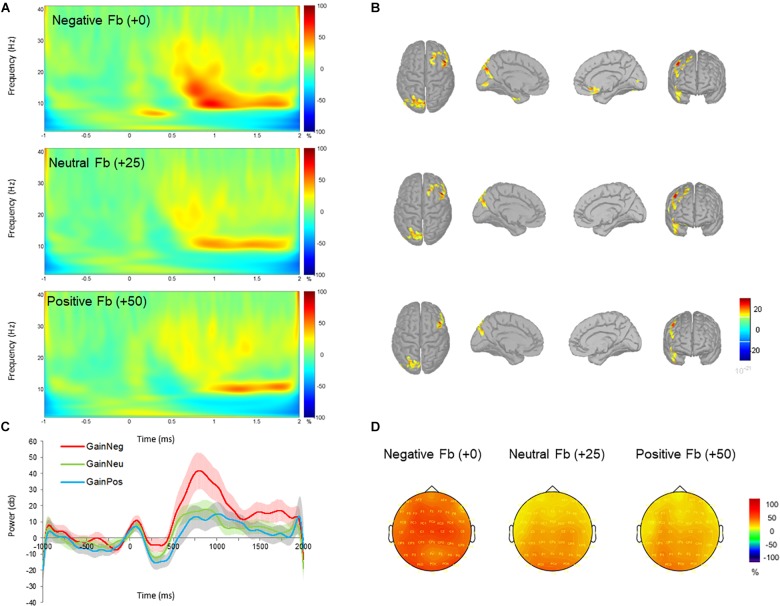
Time-frequency power (total) across negative (+0 MU), neutral (+25 MU) and positive (+50 MU) feedback for gain blocks. **(A)** Time-frequency plots at channel FCz displaying the changes in power from 700 to 1000 ms with respect to the pre-stimulus baseline (–200 to 0 ms). **(B)** Beta (12–20 Hz) source activity corresponding to each feedback type displayed for top, left medial, right medial and frontal views. Source activation maps are based on a minimum of 30 vertices with an amplitude threshold value is set to 30%. **(C)** Time-course of mean beta power with standard error bars in negative (red), neutral (green), and positive (blue) feedback conditions. **(D)** Scalp topographies plotted at 800 ms post-feedback for 15 Hz.

**FIGURE 4:**
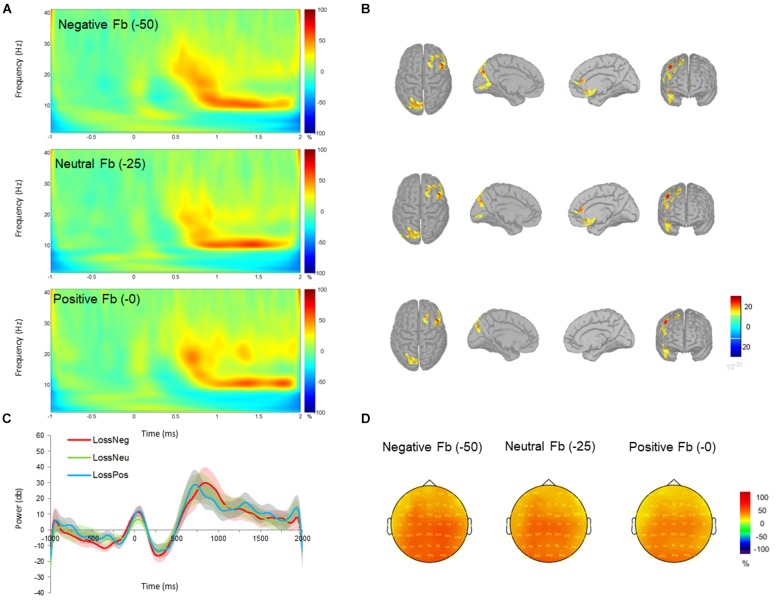
Time-frequency power (total) across negative (–0 MU), neutral (–25 MU) and positive (–50 MU) feedback for loss blocks. **(A)** Time-frequency plots at channel FCz displaying the changes in power from 700 to 1000 ms with respect to the pre-stimulus baseline (–200 to 0 ms). **(B)** Beta (12–20 Hz) source activity corresponding to each feedback type displayed for top, left medial, right medial and frontal views. Source activation maps are based on a minimum of 30 vertices with an amplitude threshold value is set to 30%. **(C)** Time-course of mean beta power with standard error bars in negative (red), neutral (green), and positive (blue) feedback conditions. **(D)** Scalp topographies plotted at 800 ms post-feedback for 15 Hz.

Unexpectedly, a late low beta (12–20 Hz) frequency component during the feedback display between 700 and 1000 ms was shown. Individual scores for beta power are shown in Figure 2B. Beta oscillations were significantly greater in power during the negative feedback condition in gain blocks. This was reflected in a three-way interaction effect between *valence, feedback*, and *electrode* which had a moderate effect size (*F*_16,384_ = 2.481, *p* = 0.001, and partial η^2^ = 0.094). *Post hoc* comparisons revealed a significant increase in beta power during processing of negative feedback as compared to positive and neutral feedback for gain blocks from all fronto-central electrode positions (all *p* < 0.05) but not CPz, CP1, and CP2. No differences were observed between neutral and positive feedback in the gain domain and no differences were observed across feedback conditions within the loss domain (all *p* > 0.05). Overall, these results suggest that changes in beta power oscillations have a specific role for gains, particularly during the omission of gains. See Figures 3, 4 for time-frequency maps, separated across gains and losses.

The repeated measures ANOVA also revealed a statistical significant moderate effect of *feedback* on beta power (*F*_2,48_ = 4.868, *p* = 0.012, and partial η^2^ = 0.169), indicating an increase in mean power of beta oscillations for negative compared to neutral feedback (*p* = 0.044) and positive feedback (*p* = 0.029). No other differences were observed between neutral and positive feedback (*p* = 0.530). The interaction effect between *electrode* and *feedback* (*F*_16,384_ = 3.639, *p* < 0.001, and partial η^2^ = 0.132) was significant, demonstrating greater oscillatory power for negative feedback compared to neutral and positive feedback at electrodes FCz, FC2, Cz, C1, and C2.

To further support the claim that beta power oscillations were specific to the omission of gains, we tested the differences in beta frequency power across gains and losses producing no monetary value (i.e., +0 MU for gains versus -0 MU for losses). This contrast allowed us to deduce whether beta oscillations were sensitive to gain omission, and not necessarily to the monetary value. A direct comparison between negative feedback during gain blocks (+0 MU) and positive feedback during loss blocks (-0 MU) relieved a two-way interaction effect between *feedback* and *electrode* (*F*_8,192_ = 3.279, *p* = 0.002, and partial η^2^ = 0.120). *Post hoc* corrections revealed greater beta power from negative feedback (-0 MU) in the gain context compared to positive feedback in loss context for electrodes FCz, FC2, Cz, C1, C2, and CPz. Overall, this finding demonstrated greater beta power for “no gains” (+ 0 MU) compared to “no losses” (-0 MU). In addition, the main effect of *feedback* was significant (*F*_1,24_ = 5.488, *p* = 0.028, and partial η^2^ = 0.186) with greater beta power for negative feedback in the gain context compared to positive feedback in the loss context (*p* = 0.028).

We next determined whether there were differences between negative feedback across gain and loss contexts (i.e., +0 MU for gains versus -50 MU for losses). No differences were observed for this contrast. Finally, we compared neutral feedback across domains (i.e., +25 MU for gains versus -25 MU for losses). This contrast also revealed no significant effects.

For all feedback conditions beta oscillations were localized to the right frontal cortex, left parietal cortex, and medial frontal structures, possibly overlapping with the medial frontal cortex and the striatum. These source estimations seem to correspond with prior lesion studies (Pujara et al., 2015) and fMRI studies (Wrase et al., 2007; Pedroni et al., 2011) and when comparing the reception and omission of gains and losses.

### Beta Oscillations and Risky Decisions

Since beta oscillations were specific to gain blocks, corresponding to previous studies showing an increase in beta power during gains compared to losses (Cohen and Ranganath, 2007; Marco-Pallerés et al., 2008, 2015; Cunillera et al., 2012; HajiHosseini et al., 2012), we aimed to perform a series of GLMs to predict whether beta power in the current trial (t) can predict the selection of risky decisions in the following trial (t + 1) within gain (Table 1A) and loss blocks (Table 1B). The rationale for this analysis is that if beta power density on the current trial can predict an increasing trend to select risky decisions in the following trial yet specifically for gain blocks, then perhaps changes in beta oscillations may shed light on the differences between decision making within gain and loss blocks. GLMs were performed with *neutral* feedback as a reference variable for positive and negative feedback. For comparison, two additional GLMs were performed using *negative* feedback as a reference variable for feedback, separately for gains and losses (see Tables 2A,B). This statistical procedure was performed to cross-check the validity of the GLM analysis, as was done in a previous study (see Yaple et al., 2017).

First of all, our results reveal main effects of positive and negative feedback for both GLMs reflecting gain and loss blocks. Within the gain blocks, positive compared to neutral feedback (β = 0.271; *p*′ = 4.32 × 10^-4^), and negative compared to neutral feedback (β = 0.246; *p*′ = 0.002) predicted risky decisions in the next trial (Table 1A). For the loss blocks, positive compared to neutral feedback (β = 0.606; *p*′ = 1.8 × 10^-15^), and negative compared to neutral feedback (β = 0.494; *p*′ = 9.18 × 10^-13^) predicted risky decisions in the next trial (Table 1B). These effects may suggest that risky decisions (positive and negative feedback) promote the tendency to select risky decisions in the next trial.

Furthermore, GLMs representing gain blocks (Tables 1A, 2B) revealed significant interaction effects between *beta power density* ×*positive* feedback. This is confirmed in both the GLM using neutral feedback as a reference variable (β = -0.390; *p*′ = 9.45 × 10^-6^; Table 1A) and the GLM using negative feedback as a reference variable (β = -0.260; *p*′ = 0.036; Table 2A). This interaction effect suggests that a *decrease* in beta power during positive feedback corresponds to an *increase* of number of risky decisions in the following trial.

In summary, power of beta oscillations increased during gain omission and predicted a decrease in risky taking in next trials within the gain blocks. Taken together, beta oscillations may signify a reward learning mechanism which modulates future decisions. Perhaps this learning mechanism plays a specific role in risky decision making in the context of uncertain gains.

## Discussion

Previous studies have revealed a mid-frontal beta oscillatory activity between 20 and 35 Hz elicited by gain compared to loss outcomes (Cohen and Ranganath, 2007; Marco-Pallerés et al., 2008; Cunillera et al., 2012; HajiHosseini et al., 2012). Marco-Pallerés et al. (2015) suggested that beta oscillatory activity underlies the cross-talk between reward, memory and attention processes following rewarding events. The aim of this research was to further test the specificity of beta oscillations to gains.

We recorded EEG while participants performed a task that yielded reception and omission of monetary incentives separately for gains and losses. Rather than demonstrating a high beta component, the results demonstrated a significant moderate effect of late low beta band (12–20 Hz) for negative feedback in the gain context, but not for the loss context. Specifically, when participants selected risky gambles a significant increase in beta power during the omission of gains (negative feedback) compared to the reception of gains (positive feedback) was found. This increase in beta power during the omission of gains was also significant when compared to reception of gains after selecting the safe option (neutral feedback).

Additional analysis was performed to test whether beta oscillations during the omission of gains differed from the omission of losses; i.e., we compared neural responses to omission of gain (+0 MU) and loss blocks (-0 MU; see Holroyd et al., 2004; Nieuwenhuis et al., 2005 for a similar approach). This analysis revealed a significant increase in beta power during the omission of gains compared to the omission of losses. We independently tested whether beta activity during negative feedback in the gain domain was significantly different compared to negative feedback in the loss domain. This contrast revealed no effect of valence or feedback. Finally, we also compared neutral feedback under gains and losses. This contrast revealed significantly greater power in losses compared to gains, which corroborates the low power of beta oscillations in the gain-neutral feedback condition.

Supplementary to the time-frequency analysis, we calculated source estimates of the low late beta component. Beta oscillations across all conditions were localized within the right prefrontal cortex, medial frontal cortex, left parietal cortex, and the striatum, corresponding to previous studies (Wrase et al., 2007; Pedroni et al., 2011; Pujara et al., 2015), which suggests that beta oscillations in the current study were not exclusively related to motor activity. Overall, our results support specificity of low late beta oscillations for processing of gains yet emphasize that beta-activity may be associated with the omission of gains.

An important distinction between the current results and prior studies relate to the spectral and temporal counterparts of beta oscillations. In the current study, beta oscillations were relatively low in frequency (12–20 Hz) and late in time (700–1000 ms) compared to previous studies (Marco-Pallerés et al., 2008, 2015; HajiHosseini et al., 2012; Leicht et al., 2013; Mas-Herrero et al., 2015; see Luft, 2014 for review). To date, only few studies investigating feedback processing have reported an increase in low beta power at around 800 ms (HajiHosseini et al., 2012; Leicht et al., 2013; Luft, 2014; Pornpattananangkul and Nusslock, 2016; Novikov et al., 2017). For example, when comparing low to high probable rewards HajiHosseini et al. (2012) revealed an increase in low beta power between 700 and 000 ms, resembling a similar pattern of activity in the current experiment. In another study, late low beta oscillations increased in power during the ‘no-reward’ compared to a ‘reward’ condition using a delayed discounting task (Pornpattananangkul and Nusslock, 2016).

Although no explanation has been provided to explain the functional role of this late beta frequency component, others have offered the possibility that multiple beta frequency components may co-occur during feedback processing (Luft, 2014). For example, Luft (2014) suggest that an additional beta component between 17 and 24 Hz may reflect a learning mechanism that orchestrates sensorimotor processing in response to errors by strengthening responses associated with wins and weaken responses associated with losses. However, it is unlikely that low beta oscillations in the current study are strictly attributed to sensorimotor processing since they were localized to the right frontal and left parietal regions, corresponding to the topographic distribution of a late beta frequency component at around 15 Hz after losses (Leicht et al., 2013).

Secondly, the source localization of the current study showing activity within the right lateralized frontal area corresponds with high-beta oscillations in an earlier study (HajiHosseini and Holroyd, 2015a), which may indicate that high and low frequency oscillations reflect intersecting oscillating processes. Modeling studies have suggested that low beta might be the result of cross-frequency interactions between high beta and gamma oscillations (Kramer et al., 2008; also see Roopun et al., 2008). Finally, while in previous studies the monetary gambling task was used to induce positive (reward) and negative (loss) feedback, our experiment used a novel task design that induces positive and negative feedback separately across gain and loss blocks by means of a risk-taking component. Due to the differences in study designs it is unclear whether our results reveal similar or different mechanism as prior studies reveal. Hence, further testing is necessary to explore the role of the late low beta component.

Importantly, probability, expected value, and magnitude of outcomes remained constant throughout the entire experiment and thus cannot account for the increase of beta oscillatory activity observed during the omissions of gains. This finding corresponds with the previous study demonstrating no change in beta power synchrony under manipulations of probability and expected value (see HajiHosseini and Holroyd, 2015b). Instead, we suggest that changes in beta power may reflect the subjective experience of relative feedback in which the omission of gains is compared in hindsight to other potential outcomes. This interpretation coincides with a recent fMRI study that demonstrated successful avoidance of losses is processed as a positive value because this value is computed relative to the value of its choice context (Palminteri et al., 2015). In contrast, an unsuccessful event in the gain domain (reward omission) may be valued as a negative monetary value since this value is computed relative to other alternative outputs. Therefore, higher power of late low beta component may be functionally related to negative monetary value. This premise accounts for our results since the power of beta oscillations were most robust for feedback conditions in which no monetary outcome was rewarded (+0, -0, -25, and -50).

To explore the functional role of beta oscillations on risky decision making, we also investigated whether beta power density on each trial would predict the tendency to select risky decisions on the following trial. The GLM predicting risky decision making in the following trial demonstrated an interaction effect between beta oscillatory power and positive feedback, yet specifically for gain blocks. The relationship between the interaction (beta PSD × positive feedback) and risky decision making was negative (i.e., β = -0.390), suggesting that during positive feedback a *decrease* in beta oscillatory power reflects an *increase* in risky decision making in the following trial. This suggests that the reduction in beta power during the negative feedback display motivates one to select risky decisions in following trials.

To interpret this result, we propose a reward learning mechanism marked by changes in beta oscillations between trials. When receiving positive feedback, an increase in beta power reinforces the decision maker to continue to select risky gambles. However, during the absence of gains, a violation of rewards occurs in which the gain omission relative to alternative prospective outcomes results to an increase in beta oscillatory power as the result of perceiving gain omission as a “loss” (see Palminteri et al., 2015 for more details). In turn, this reward violation decreases the tendency to select future risky gambles. Perhaps this reward learning mechanism concurs with the anticipatory affect model of choice proposed by Knutson and Greer (2008). The anticipatory affect as marked by various events (including unexpected positive versus negative events) influences subsequent choice.

Perhaps this proposed reward learning mechanism may also explain the observed results in a prior experiment in which induced 20 Hz transcranial electric current stimulation increased risky decision making (Yaple et al., 2017). As the result of perturbing the endogenous beta oscillations underlying this mechanism, participants had not experienced a sense of reward violation, and henceforth overcompensated by increasing the tendency to select risky gambles.

## Conclusion

In the current study we showed that late low beta oscillations between 12 and 20 Hz are functional sensitive to gain omission relative to other potential gains. Furthermore, beta oscillations elicited by positive feedback in the gain domain were negatively associated with risky decision making in the following trial. From these two novel findings, we propose a reward learning mechanism by which the power of beta oscillations manifested by outcome violation, motivates responders to change subsequent choices as a means to compensate for reward omission on the current trial. We further contend that due to the novelty of this finding, further work is necessary to determine whether late low beta oscillations reflect a similar or alternative feedback-related beta component reported in the high beta range.

## Author Contributions

ZY, AS, and VK designed the research and wrote the paper. ZY performed the research. ZY, MM-S, NN, and DA analyzed the data.

## Conflict of Interest Statement

The authors declare that the research was conducted in the absence of any commercial or financial relationships that could be construed as a potential conflict of interest. The reviewer CK and handling Editor declared their shared affiliation.

## Footnotes

1 http://neuroimage.usc.edu/brainstorm
