# Supplementary material for: Power of Feedback-Induced Beta Oscillations Reflect Omission of Rewards: Evidence From an EEG Gambling Study
Source: Front Neurosci. 2018 Oct 30;12:776. doi: 10.3389/fnins.2018.00776 (PMC6218571; doi:10.3389/fnins.2018.00776)
Supplement: Supplementary file 1 [file Data_Sheet_1.docx]

Supplementary materials for:

Power of feedback-induced beta oscillations reflect omission of rewards: evidence from an EEG gambling study

Zachary Yaple, Mario Martinez-Saito, Nikita Novikov, Dmitrii Altukhov, Anna Shestakova, & Vasily Klucharev

*Region of interest (ROI) selection:*

Using a data-driven approach relying on the ERSP data we selected a ROI for the main analysis. The ROI selection procedure was based on the full-dimensional (time x frequency x channel) ERSP data, without a priori selection of electrodes. The ERSP was calculated using a pre-response baseline of -200 - 0 ms, with averaging across all experimental conditions. The temporal, spectral and spatial window of the ROI was assessed by performing a statistical comparison of the ERSP data against zero. Please note that the statistical analysis of the ERSP is orthogonal to our main cross-condition analysis.

The statistical comparison was performed as follows. First, we considered each data bin (time x frequency x channel) separately, selected the vector of ERSP values at this data bin for all subjects, and compared this vector with zero using t-test. Repeating this procedure for each data bin, we obtained a map (time x frequency x channel) of t-scores. Next, we transformed this map using the TFCE (threshold-free cluster enhancement) algorithm (see Novikov et al., 2015, 2017 for prior examples; also see Smith and Nichols, 2009). This algorithm increases the absolute values of t-scores for those data bins that fall into “clusters” and suppresses random statistical fluctuations of the t-scores. As a result, we obtained a map (time x frequency x channel) of TFCE-scores. After this, we repeated the whole procedure on permuted data. At each permutation step, we randomly selected a subset of subjects and flipped the sign of the ERSP data (for all data bins at the same time) for those subjects. Then we performed the t-test and the TFCE (as it was describe above), and obtained a permuted TFCE map. From this map, we chose the minimal and the maximal value. Repeating this for 1000 permutation steps, we obtained distribution of the maximal permuted TFCE-scores and distribution of the minimal TFCE-scores. Finally, we compared the unpermuted TFCE-score in each data bin with these two distributions, thus obtaining the p-value for this bin. Repeating this for all bins, we obtained a map (time x frequency x channel) of p-values.

The result is presented in the Supplementary Figure 1. It clearly demonstrates a centrally located ERSP cluster (p < 0.001) in the beta band. Based on the obtained results, we selected the group of central electrodes for the ROI: FCz, FC1, FC2, Cz, C1, C2, CPz, CP1, and CP2. We also selected the frequency band (12 - 20 Hz) and time interval (700 - 1000 ms) for the ROI.


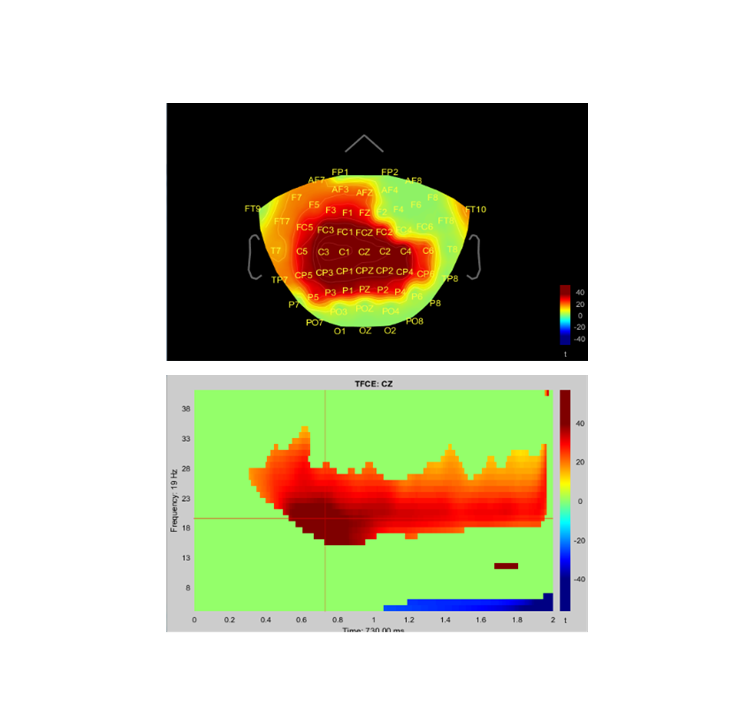


**Supplementary Figure 1**: represents the ERSP averaged across subjects, thresholded at p<0.001. The upper panel represents the ERSP topography at 20 Hz and 750 ms; the lower panel represents the time-frequency plot for the Cz electrode.

| Supplementary Table 1a. GLM for rewards with neutral feedback as reference | | | | |  | |  |  |  |  |  |
| --- | --- | --- | --- | --- | --- | --- | --- | --- | --- | --- | --- |
|  | β | SE | z-value | p-value | p’ |  |  |  |  |  |  |
| Beta PSD | 0.073 | 0.041 | 1.750 | 0. 080 | 0.080 |  |  |  |  |  |  |
| **Fb (+50)** | **0.245** | **0.066** | **3.684** | **2.2x10^-4^** | **6.8x10^-4^** |  |  |  |  |  |  |
| **Fb (+0)** | **0.266** | **0.066** | **3.985** | **6.7x10^-5^** | **2.6x10^-4^** |  |  |  |  |  |  |
| **Beta PSD*Fb (+50)** | **-0.349** | **0.072** | **-4.789** | **1.68x10^-6^** | **8.4x10^-6^** |  |  |  |  |  |  |
| Beta PSD*Fb (+0) | -0.142 | 0.067 | -2.094 | 0.036 | 0.072 |  |  |  |  |  |  |

| Supplementary Table 1b. GLM for losses with neutral feedback as reference | | | | |  | |  | |  |  |  |  |
| --- | --- | --- | --- | --- | --- | --- | --- | --- | --- | --- | --- | --- |
|  | β | SE | z-value | p-value | | p’ | |  |  |  |  |  |
| Beta PSD | 0.075 | 0.044 | 1.694 | 0.090 | | 0.451 | |  |  |  |  |  |
| **Fb (-0)** | **0.609** | **0.065** | **9.262** | **<2x10^-16^** | | **1x10^-15^** | |  |  |  |  |  |
| **Fb (-50)** | **0.491** | **0.066** | **7.406** | **1.3x10^-13^** | | **6.5x10^-13^** | |  |  |  |  |  |
| Beta PSD*Fb (-0) | -0.141 | 0.072 | -1.960 | 0.049 | | 0.249 | |  |  |  |  |  |
| Beta PSD*Fb (-50) | -0.118 | 0.062 | -1.911 | 0.056 | | 0.280 | |  |  |  |  |  |

Note: β = Beta coefficient represent standardized effect sizes; SE = Standard error of the mean; z-value based on Wald test; PSD = Power Spectral Density; p’ = corrected p value; Fb = Feedback; Bold font indicates statistical significance after Holm-Bonferroni correction

**Supplementary Table 1.** Generalized Logistic Model (GLM) [theta PSD predictor omitted] predicting risk decision making in the following trial for rewards (a) and losses (b) with neutral feedback as the reference variable. Spectral power density was extracted from each trial between 12-20 Hz (Beta PSD).

| Supplementary Table 2a. GLM for rewards with negative feedback as reference | | | | | |  | |  |  |  |  |  |
| --- | --- | --- | --- | --- | --- | --- | --- | --- | --- | --- | --- | --- |
|  | β | SE | z-value | p-value | p' | |  |  |  |  |  |  |
| Beta PSD | -0.068 | 0.061 | -1.128 | 0.259 | 0.518 | |  |  |  |  |  |  |
| Fb (+50) | -0.020 | 0.070 | -0.289 | 0.772 | 0.772 | |  |  |  |  |  |  |
| **Fb (+25)** | **-0.266** | **0.066** | **-3.985** | **6.7x10^-5^** | **3.3x10^-4^** | |  |  |  |  |  |  |
| **Beta PSD*Fb (+50)** | -0.207 | 0.083 | -2.481 | 0.013 | 0.052 | |  |  |  |  |  |  |
| Beta PSD*Fb (+25) | 0.142 | 0.067 | 2.094 | 0.036 | 0.108 | |  |  |  |  |  |  |

| Supplementary Table 2b. GLM for losses with negative feedback as reference | | | | | |  |  |  |  |  |
| --- | --- | --- | --- | --- | --- | --- | --- | --- | --- | --- |
|  | β | SE | z-value | p-value | p' |  |  |  |  |  |
| Beta PSD | -0.042 | 0.048 | -0.888 | 0.374 | 0.748 |  |  |  |  |  |
| Fb (-0) | 0.118 | 0.062 | 1.911 | 0.100 | 0.300 |  |  |  |  |  |
| **Fb (-25)** | **-0.491** | **0.066** | **-7.406** | **1. 3x10^-13^** | **6.5x10^-13^** |  |  |  |  |  |
| Beta PSD*Fb (-0) | -0.023 | 0.076 | -0.303 | 0.761 | 0.761 |  |  |  |  |  |
| Beta PSD*Fb (-25) | 0.118 | 0.062 | 1.911 | 0.055 | 0.223 |  |  |  |  |  |

Note: β = Beta coefficient represent standardized effect sizes; SE = Standard error of the mean; z-value based on Wald test; PSD = Power Spectral Density; p’ = corrected p value; Fb = Feedback; Bold font indicates statistical significance after Holm-Bonferroni correction

**Supplementary Table 2.** Generalized Logistic Model (GLM) [theta PSD predictor omitted] predicting risk decision making in the following trial for rewards (a) and losses (b) with negative feedback as the reference variable. Spectral power density was extracted from each trial between 12-20 Hz (Beta PSD).
